# Supplementary material for: Engineering of CD19 Antibodies: A CD19-TRAIL Fusion Construct Specifically Induces Apoptosis in B-Cell Precursor Acute Lymphoblastic Leukemia (BCP-ALL) Cells In Vivo
Source: J Clin Med. 2021 Jun 15;10(12):2634. doi: 10.3390/jcm10122634 (PMC8232684; doi:10.3390/jcm10122634)
Supplement: Supplementary file 1 [file jcm-10-02634-s001.zip › jcm-1223890-supplementary.pdf]

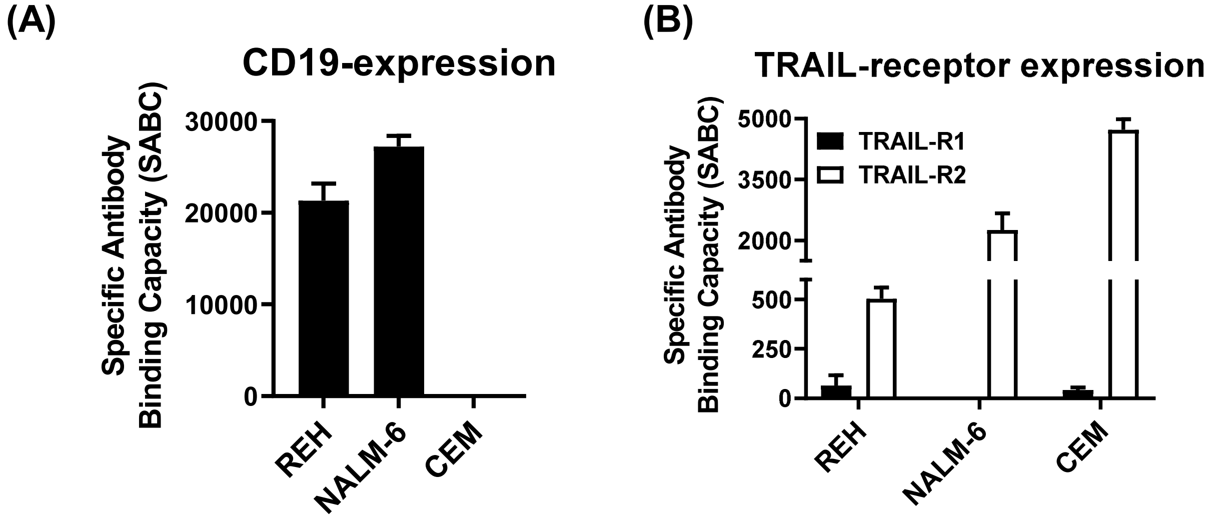

Supplementary Figure S1. Expression profile of CD19 and TRAIL receptors (TRAIL-R1/TRAIL-R2) on leukemic cell lines. A-B) Surface expression of (A) CD19 or (B) TRAIL-R1 or TRAIL-R2 was quantified using the QIFIKIT® (Agilent Technologies). Data show mean values ± SEM of n=3 independent experiments .

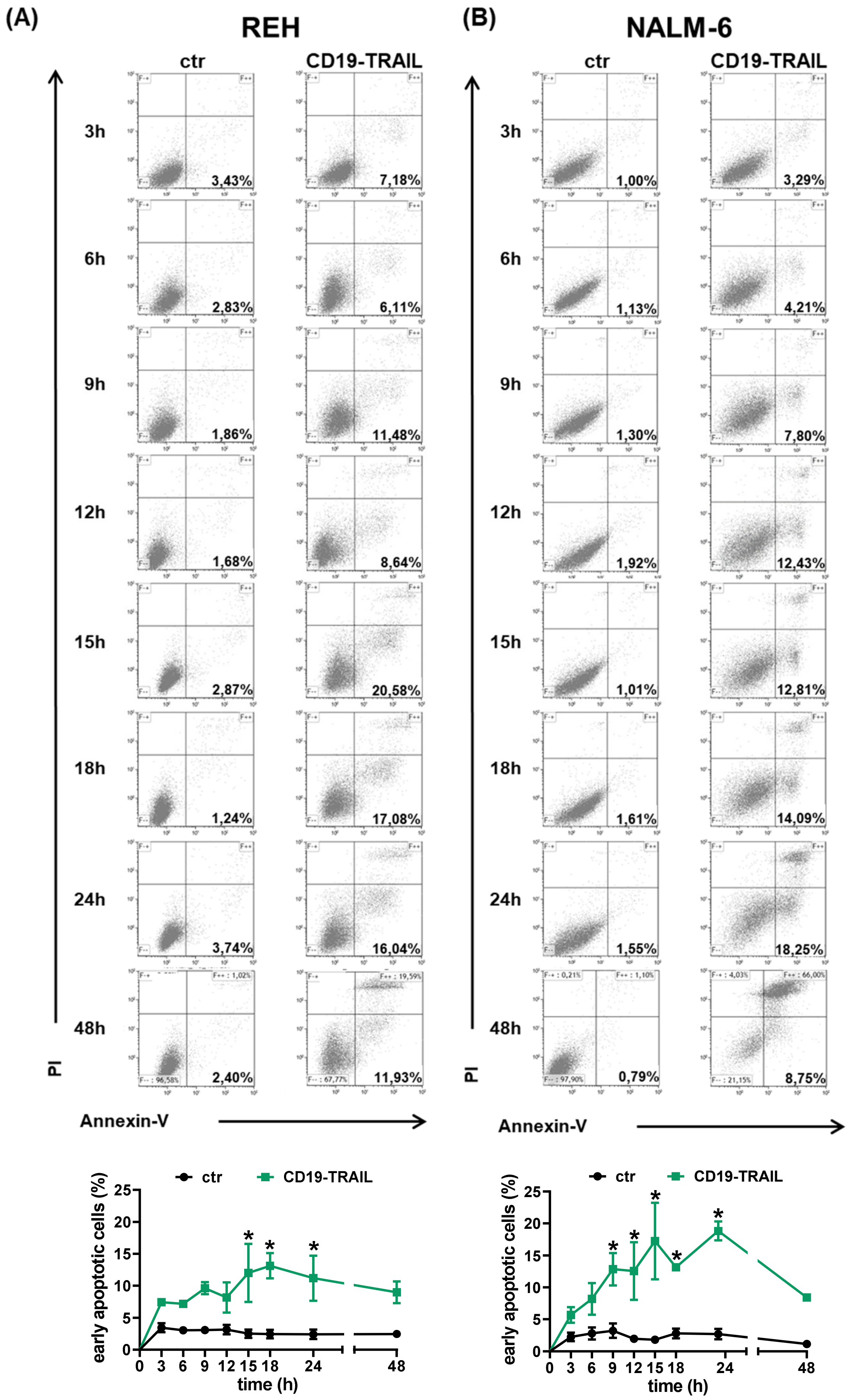

Supplementary Figure S2. CD19-TRAIL kills ALL-cells via apoptosis induction A-B) REH and NALM-6 cells were treated for indicated periods with CD19-TRAIL or left untreated (ctr). A) REH cells and B) NALM-6 cells were examined for early apoptotic (Annexin$^+$/PI$^-$) and late apoptotic (Annexin$^+$/PI$^+$) cell populations by flow cytometry. Representative flow cytometry plots as well as mean values ± SEM of n=3 independent experiments are shown, respectively. Two way analysis of variance, *P<0.05.

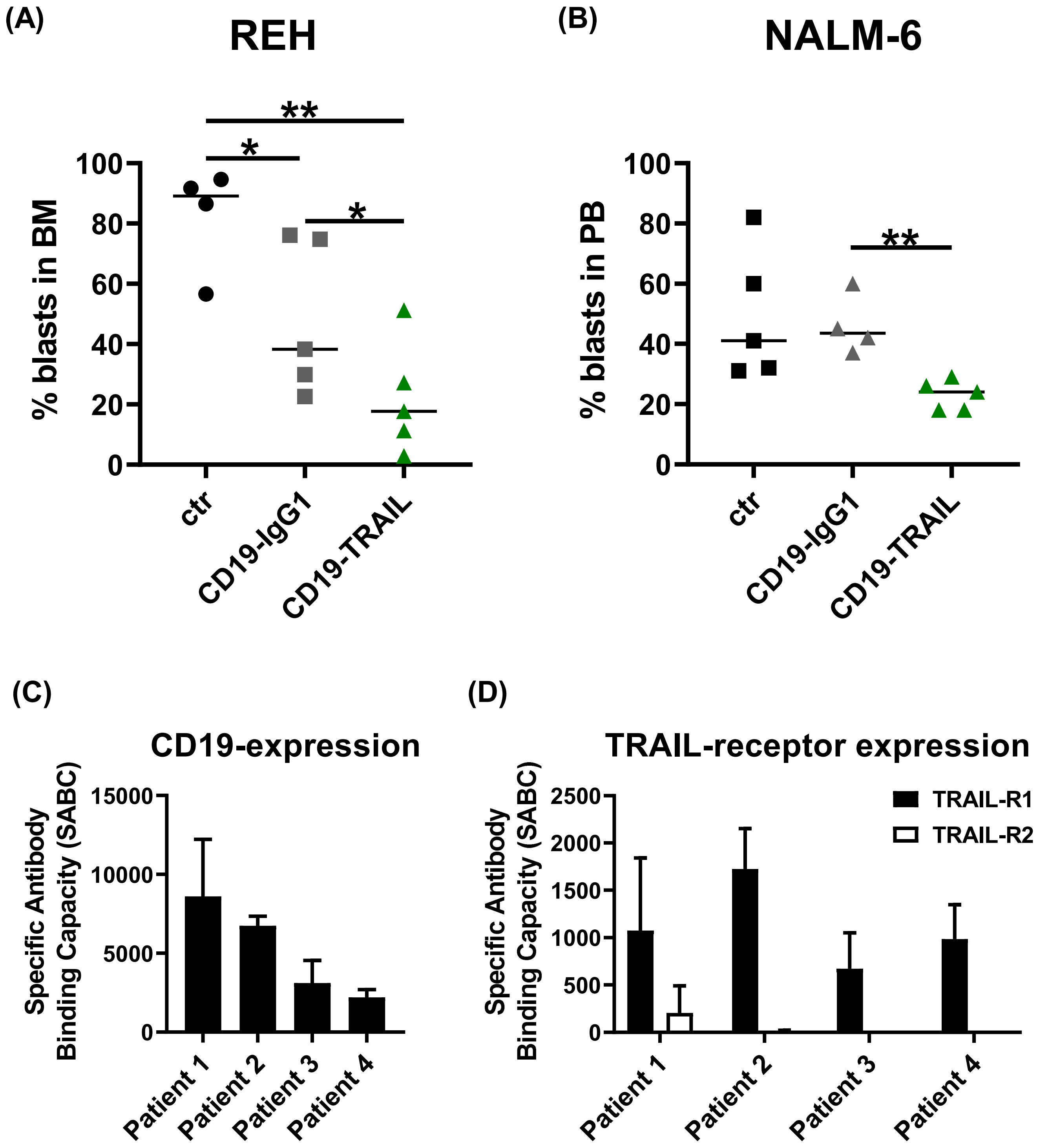

Supplementary Figure S3. CD19-TRAIL eradicates BCP-ALL cells in vivo. A-B) NSG mice were injected with 5*10^5 REH or NALM-6 cells (n=5 per group) (day 0). On days+1, +3 ,+6 , +10 ,+13 and every 7 days thereafter mice were treated with 1.5 mg/kg of CD19-Ig G1 or CD19-TRAIL intravenously or left untreated (ctr). A) On day +32, engraftment of REH cells was assessed via flow cytometry detection of hCD45+/mCD45-cells in bone marrow (BM) aspirates. Depicted are relative leukemia cell counts of n=5 animals, one-tailed Mann-Whitney-Test. B) Relative counts of leukemic cells in the peripheral blood (PB) of animals injected with NALM-6 cells and treated with CD19-IgG1 or CD19-TRAIL as of day 21 post injection and ctr animals upon sacrifice (day 17). Due to the lack of CD45 in NALM-6 cells and blocking of the CD19 epitope by CD19-IgG1 or CD19-TRAIL, *in vivo* eng raftment of NALM-6 cells was assessed via morphological differential blood count analysis. Depicted are relative leukemia cells counts of n=5 animals, one-tailed Mann-Whitney-Test. *P<0.05, **P<0.01. C-D) Surface expression of (C) CD19 or (D) TRAIL-R1 or TRAIL-R2 was quantified using the QIFIKIT® (Agilent Technologies). Data show mean values ± SEM of n=3 independent experiments.

(A)

CD19-TRAIL [nM]	Venetoclax [nM]	Effect (%)	CI Value
1,5	150	99	0.27692
0,3	30	95	0.25490
0,06	6	81	0.23330
0,012	1,2	46	0.27403
0,0024	0,24	13	2.23561

(B)

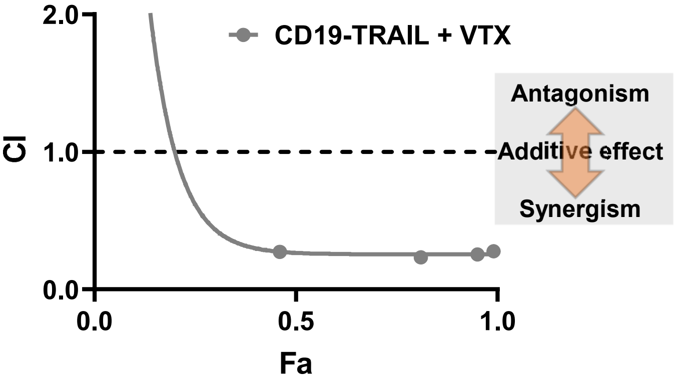

Supplementary Figure S4. ALL-killing effect of CD19-TRAIL is synergistically enhanced by Venetoclax A) REH cells were treated with escalating concentrations of CD19-TRAIL, the BCL-2 inhibitor Venetoclax (VTX) or the combination of both. The combination index (CI) values for each individual combination data point were calculated. CI<1 represents synerg ism, CI=1 indicates additive effect and CI>1 represents antag onism. B) CI plot g enerated by Compusyn Software where the CI is plotted as a function of the fraction affected (Fa) for the additive anti-proliferative effects of CD19-TRAIL and VTX. Data are presented as mean values from n = 3 individual experiments.

Supplementary Table S1. Clinical characteristics of BCP-ALL patients xenografted in NSG mice

Patient Data							Xenograft Data				
**Patient**	**Age (y)**	**Sex**	**WBC**	**MRD Risk**[1]	**PR**[2]	**Cytogenetic**	**% BM blasts**	**Survival (days)**	**CD19 (SABC)**	**TRAIL-R1 (SABC)**	**TRAIL-R2 (SABC)**
1	16.9	F	≥10.000 <50.000	HR	G	BCR-ABL+	98, 99	124, 73	8603	1076	206
2	9.4	F	≥100.000	SR	G	BCR-ABL+	96,99	141, 154	6738	1726	0
3	10.3	M	<10.000	IR	G	E2A-PBX1+	92,94	53, 53	3125	673	0
4	0.3	F	≥100.000	N/A	G	MLL-rearranged	99, 87	111, 50	2210	985	0

[1]Risk stratification according to MRD risk groups: MRD-SR: TP1+2 negative, MRD-IR: TP1 and/or TP2 $<10^{-3}$, MRD-HR: TP2 $\geq 10^{-3}$.
[2]PR: Prednisone response; G: good (less than 1000 leukemic blasts/µl blood on treatment day 8); P: poor (more than 1000/µl on day 8)
N/A Data not available
WBC: White blood cell count at initial diagnosis
SABC: Specific antibody binding capacity
